# A Systematic Review on Prostate-Specific Membrane Antigen Positron Emission Tomography (PSMA PET) Evaluating Localized Low- to Intermediate-Risk Prostate Cancer: A Tool to Improve Risk Stratification for Active Surveillance?

**DOI:** 10.3390/life14010076

**Published:** 2024-01-02

**Authors:** Jianliang Liu, Jordan Santucci, Dixon T. S. Woon, Rick Catterwell, Marlon Perera, Declan G. Murphy, Nathan Lawrentschuk

**Affiliations:** 1 EJ Whitten Prostate Cancer Research Centre, Epworth Healthcare, Melbourne, VIC 3005, Australia; 2 Department of Urology, The Royal Melbourne Hospital, University of Melbourne, Melbourne, VIC 3052, Australia; 3 Department of Surgery, University of Melbourne, Melbourne, VIC 3052, Australia; declan.murphy@petermac.org; 4 Department of Surgery, Peter MacCallum Cancer Centre, Melbourne, VIC 3052, Australia; 5 Department of Urology, University of Melbourne, Austin Health, Heidelberg, VIC 3084, Australia; 6 Department of Urology, The Queen Elizabeth Hospital, Woodville, SA 5011, Australia; 7 Discipline of Surgery, University of Adelaide, Adelaide, SA 5005, Australia

**Keywords:** active surveillance, prostate cancer, prostate-specific membrane antigen positron emission tomography/computed tomography

## Abstract

Active surveillance remains a treatment option for low- to intermediate-risk prostate cancer (PCa) patients. Prostate-specific membrane antigen positron emission tomography and computed tomography (PSMA PET/CT) has emerged as a useful modality to assess intraprostatic lesions. This systematic review aims to evaluate PSMA PET/CT in localized low- to intermediate-risk PCa to determine its role in active surveillance. Following PRISMA guidelines, a search was performed on Medline, Embase, and Scopus. Only studies evaluating PSMA PET/CT in localized low- to intermediate-risk PCa were included. Studies were excluded if patients received previous treatment, or if they included high-risk PCa. The search yielded 335 articles, of which only four publications were suitable for inclusion. One prospective study demonstrated that PSMA PET/CT-targeted biopsy has superior diagnostic accuracy when compared to mpMRI. One prospective and one retrospective study demonstrated MRI occult lesions in 12.3–29% of patients, of which up to 10% may harbor underlying unfavorable pathology. The last retrospective study demonstrated the ability of PSMA PET/CT to predict the volume of Gleason pattern 4 disease. Early evidence demonstrated the utility of PSMA PET/CT as a tool in making AS safer by detecting MRI occult lesions and patients at risk of upgrading of disease.

## 1. Introduction

Prostate cancer (PCa) is the second most commonly diagnosed cancer worldwide [1]. It is estimated that by 2040, there will be close to 2.3 million new cases of PCa due to an ageing population [2]. The introduction of the prostate-specific antigen (PSA) blood test has also contributed to increased detection of PCa cases, resulting in the identification of low-risk PCa becoming more common as well [3]. Active treatment (surgery or radiotherapy) for these low-risk PCa cases does not appear to improve cancer-specific survival and close observation appeared to be more beneficial [4]. Consequently, the treatment regime of active surveillance was introduced.

Active surveillance is a structured monitoring program consisting of regular PSA testing, clinical examinations, and repeat prostate biopsies when necessary [5]. The goal of active surveillance is to avoid unnecessary treatment of clinically insignificant localized PCa while detecting disease progression that may necessitate active treatment [5].

Currently, the majority of guidelines recommend active surveillance for localized low-risk or favorable intermediate-risk PCa based on the D’Amico classification [4,6,7,8]. However, there is a lack of consensus on which subgroup of intermediate-risk PCa patients is best suited for active surveillance and criteria to progress to active treatment. Some intermediate-risk PCa patients undergoing active surveillance have been shown to have worse overall survival and metastatic-free survival when compared to low-risk PCa patients [9]. Additionally, close to one-third of patients on active surveillance develop disease progression which may require active treatment [10]. Therefore, there is room for us to improve our risk stratification and selection of active surveillance patients.

The current imaging modality used to guide repeat prostate biopsies is multiparametric magnetic resonance imaging (mpMRI) [11]. Although mpMRI has greatly improved our detection of clinically significant PCa (csPCa), 13% of csPCa still gets missed [12]. Prostate-specific membrane antigen positron emission tomography (PSMA PET) has emerged as a useful modality to assess intra-prostatic cancer [13]. Intraprostatic avidity on PSMA PET appears to correlate with the grade of the intraprostatic PCa [14] and has the potential to provide prognostic information such as predicting progression-free survival [15]. When combined with MRI, PSMA PET can improve csPCa detection [16]. However, there is no systematic review to date evaluating the role of PSMA PET in the active surveillance cohort. This systematic review aims to critically appraise the existing literature which utilizes PSMA PET in localized low- to intermediate-risk PCa to determine if it could help improve risk stratification and selection of patients for active surveillance.

## 2. Materials and Methods

### 2.1. Literature Search Strategy

This systematic review was registered on PROSPERO under the registration number CRD42023449769. The Preferred Reporting Items for Systematic Reviews and Meta-analyses (PRISMA) guidelines were used. A comprehensive literature search was performed on Medline, Embase, and Scopus for publications from inception until June 2023. A combination of key search terms used include: (“prostate-specific membrane antigen” OR “PSMA”) and (“positron emission tomography” OR “PET”) and (“active surveillance” or “low-risk prostate neoplasm” or “intermediate-risk prostate neoplasm”).

### 2.2. Eligibility Criteria

The population, intervention, comparator, and outcome (PICO) system was used to guide our eligibility criteria. To closely mimic patients who may be suitable for active surveillance, this systematic review only included patients with biopsy-confirmed low- to intermediate-risk prostate cancer according to the D’Amico classification [17]. Low-risk PCa patients were defined as patients with International Society of Urologic Pathology Grade Groups (ISUP GG) 1, and PSA ≤ 10 ng/mL. Intermediate-risk PCa patients were defined as ISUP GG 2 to 3 and/or PSA between 10 to 20 ng/mL. The intervention of interest was the use of a PSMA PET scan in low- to intermediate-risk PCa to assess intraprostatic lesions. The PSMA PET scan had to be performed prior to active treatment or during active surveillance. All variations of PSMA tracers were included (e.g., 18F-DCFPyL PSMA or 68Ga-PSMA). Comparison of PSMA PET results may be made against MRI or between ISUP GG. In studies assessing PSMA PET results of patients with pathological upstaging, the comparison will be made against PSMA PET results of patients without pathological upstaging. The maximum standardized uptake value (SUVmax) was defined as the region with the highest PSMA uptake activity. The primary endpoint of this systematic review is to determine if a PSMA PET scan can improve risk stratification of low- to intermediate-risk PCa. Primary outcome measures include the ability of the PSMA PET scan to predict pathological upstaging or underlying adverse pathology (e.g., cribriform pattern), and to differentiate the percentage of Gleason pattern 4 diseases. The secondary aim of this study is to determine how PSMA PET can be incorporated into active surveillance. All English language original articles were considered.

The following types of articles were excluded: case reports, studies that included less than five cases, reviews, letters to journals, conference abstracts, and articles not written in English. Studies involving the following groups of patients were also excluded: benign biopsy, high-risk PCa (i.e., ISUP GG 4 and 5), patients with metastatic PCa, PCa patients who have previously undergone active treatment (e.g., surgery or radiotherapy) prior to PSMA PET.

### 2.3. Screening and Study Selection

Titles and abstracts were independently reviewed by two authors (J.L. and J.S.) and any disagreements in conflicts were resolved by the senior author. The full text of the remaining relevant articles was then reviewed. The only automation tool used was Covidence for the removal of duplicated articles and to assist in the screening process. Data from included papers were extracted onto an Excel spreadsheet for analysis.

### 2.4. Quality Assessment

The Quality Assessment of Diagnostic Accuracy Studies (QUADAS-2) tool was employed to evaluate the risk of bias and applicability of the included studies (see Table 1). This was done independently by two authors (J.L. and J.S.) and any conflicts were resolved by the senior author.

## 3. Results

### 3.1. Literature Search

**Figure 1:**
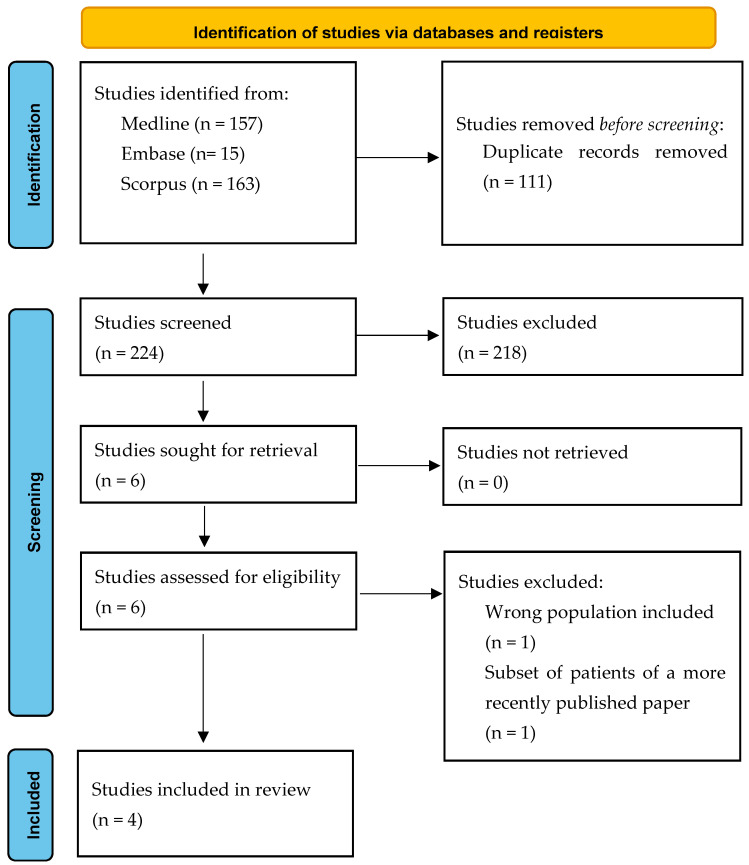
PRISMA flow diagram.

The search yielded 335 articles, of which 111 were duplicates (see Figure 1). After screening 224 titles and abstracts, six articles were sought for full-text reviews. During full-text reviews, two studies were excluded for the following reasons. The first paper was excluded as it enrolled patients without PCa (i.e., benign biopsy) and patients with high-risk PCa (i.e., ISUP GG 4 and 5) [22]. Another paper was excluded as it was an old study [23]. The updated study which enrolled more patients and provided more comprehensive results was included in this systematic review [18].

Four studies were deemed suitable for this systematic review, two of which were prospective in nature: one was a single-center study by Pepe et al. [18] and the other was a multi-center study by Heetman et al. (PASPoRT Trial, study ID NL69880.100.19) [19]. The remaining two studies were retrospective in nature: one was the experience of a single surgeon by Jain et al. [20], and another was a retrospective analysis of a prospectively collected multi-center database by Xue et al. [21].

### 3.2. Baseline Characteristics of Patients in the Included Studies

A total of 431 patients were included across the four studies. The age of the included patients ranged from 52 to 74 years old, and the PSA level ranged from 3.7 to 8.60 ng/mL (see Table 2). Overall median age and median PSA were not available for two studies. The types of patients enrolled in the included studies are as follows: Pepe et al. [18] only included low-risk PCa patients on active surveillance, Heetman et al. [19] only enrolled low- to intermediate-risk PCa patients on active surveillance, Jain et al. [20] only enrolled patients with low- to favorable intermediate-risk PCa who were recommended active surveillance as one of their treatment options, but patients may have opted for active treatment, and Xue et al. [21] enrolled all patients with biopsy or prostatectomy-proven intermediate-risk PCa. All studies used PSMA PET/CT and none used PSMA PET/MRI. Three of the studies used only a single type of tracer (68Ga PSMA). Only the study by Jain et al. [20] included scans performed with various PSMA tracers (either 68Ga-HBEDD-11 or 18F-DCFPYL). The study by Pepe et al. [18] evaluated PSMA PET scans performed five years after the confirmatory biopsy. The remaining three studies were PSMA PET scans performed after the diagnostic biopsy.

### 3.3. MRI Occult Lesions and PSMA-Targeted Biopsy

An MRI occult lesion was defined as a PSMA PET/CT avid intra-prostatic lesion that was not detected on the MRI. In the study by Jain et al. [20], four (13.3%) out of 30 patients harbored MRI occult lesions. During the sampling of these MRI occult lesions, three (75%) out of four were positive for PCa. These positive MRI occult lesions had an ISUP GG concordant with their initial diagnosis (no upstaging).

PSMA PET/CT-targeted biopsies were also explored in the study by Heetmann et al., [19] where they used a cut-off SUVmax of ≥4 not covered by previous biopsy. Forty-five (32%) out of 141 patients required additional PSMA PET/CT-targeted biopsies due to MRI occult lesions in 41 (29%) of patients and an SUVmax thought to be discordant to their initial ISUP GG in four (3%) patients. Upon PSMA-targeted biopsy, 14 (10%) out of 141 patients harbored more sinister pathology such as a cribriform pattern (n = 1) and pathological upgrading of disease (n = 13, nine ISUP GG 2, two ISUP GG 3, one ISUP GG 4, and one ISUP GG 5). Of these 13 patients with pathology upgrading, six remained under active surveillance and seven underwent active treatment (two radiotherapy and five prostatectomy). Of the five patients who underwent prostatectomy, three of them had histopathology which was concordant to the PSMA targeted prostate biopsy, two of the prostatectomy patients had histopathology downstaging as compared to the PSMA-targeted prostate biopsy (ISUP GG 3 to GG 2 and ISUP GG 4 to GG 3). However, these two patients still had histopathology upstaging when compared to their initial diagnostic biopsy (both had ISUP GG 1 before PSMA PET/CT).

The remaining two studies did not describe the number of MRI occult lesions. Notably, in the study by Pepe et al. [18], PSMA PET/CT demonstrated fewer lesions suspected to be PCa when compared to mpMRI (22.5% versus 45%). However, when performing an image-guided target biopsy of these lesions, PSMA PET/CT and mpMRI both offered similar positive rates (66.6%). This translated to PSMA PET/CT-targeted biopsies having a lower false positive rate when compared to mpMRI-targeted biopsies (17.5% versus 40%).

### 3.4. Using PSMA PET/CT to Predict Upgrades in Prostate Cancer Grade

The addition of PSMA PET scans in the study by Heetmann et al. [19] resulted in a pathological upgrading of disease in 9% of patients. With a boxplot, Heetmann et al. demonstrated a trend where increasing ISUP GG is associated with an increase in PSMA avidity (SUVmax). This phenomenon has been described in previous studies that correlated PSMA PET/CT with prostatectomy histology [24].

The study by Jain et al. [20] followed 30 men who met the criteria for active surveillance. These patients subsequently underwent a PSMA PET/CT and 15 (50%) had concerning features on the PSMA PET/CT such as elevated SUVmax > 5, MRI occult lesions, or evidence of extra-prostatic extension (EPE). These 15 patients subsequently underwent prostatectomy. Histology revealed that nine (60%) of these 15 patients had underlying adverse pathological features (upgrading of ISUP GG, EPE, cribriform pattern, or intraductal pattern).

In the study by Xue et al. [21], SUVmax increased with an increasing percentage of Gleason pattern 4 disease. The SUVmax could be used to predict the percentage of Gleason pattern 4 disease at three different thresholds: 10%, 20%, and 50% (*p* < 0.001). On receiver operating characteristic (ROC) curve analysis, SUVmax could still discriminate the percentage of Gleason pattern 4 disease at three different thresholds, with an area under the curve (AUC) of 0.74 (0.69–0.79), 0.73 (0.66–0.78), and 0.78 (95% CI 0.71–0.83), respectively. On multivariable analysis, SUVmax remained an independent predictor at all three thresholds. The threshold SUVmax of 5.4 predicted pathological upgrading with 91% specificity and a negative predictive value of 94%.

This has two clinically significant implications. The ability of SUVmax to differentiate <50% versus >50% Gleason pattern 4 disease can help distinguish ISUP GG 2 disease from ISUP GG 3 disease, which has clear implications for the patient requirement for active surveillance versus active treatment. Secondly, this could possibly make active surveillance safer by identifying ISUP GG 2 PCa patients with a higher percentage of Pattern 4 disease who may benefit from active treatment instead.

### 3.5. Risk of Bias in the Included Studies

During the QUADAS-2 assessment of the included studies, two studies had concerns of a high risk of bias and one had an unclear risk of bias (see Table 1). One of these publications was a retrospective, single-center study of patients managed by a single surgeon, which included patients who had PSMA PET scans done as staging prior to active treatment [20]. The study by Xue et al. also had a high risk of bias due to its retrospective nature, the lack of blinding, and the inclusion of patients who had PSMA PET scans done as staging prior to active treatment [21]. Additionally, the study by Xue et al. did not perform inter-scanner standardization; therefore, the calculated SUVmax may have varied between the four different imaging sites. One study had an unclear risk of bias as the selection of some patients for additional PSMA-targeted biopsies was subjectively determined by a multidisciplinary team (MDT), with 7% of patients from this study not having post-PSMA PET scan biopsy results [19].

The applicability of the study by Pepe et al. was unclear as PSMA PET scanning was only performed in patients undergoing active surveillance five years from their confirmatory biopsy, which may have systematically excluded patients with aggressive disease. It is therefore unclear how this study applies to patients who are at the commencement of their active surveillance journey [18].

## 4. Discussion

In this systematic review, we found four publications evaluating intraprostatic lesions of low- to intermediate-risk PCa using PSMA PET/CT. Collectively, the data demonstrated that PSMA PET/CT is a promising tool in risk stratifying low- to intermediate-risk PCa patients and determining their suitability for active surveillance. PSMA PET/CT has been shown to be capable of not only picking up MRI occult lesions present in up to 32% of patients but also identifying patients who are at risk of underlying sinister pathology or upstaging. The ability of PSMA PET/CT to predict upgrading of PCa grade was also confirmed in previous studies analyzing prostatectomy histopathology [15]. These at-risk patients could be offered active treatment to avoid compromising oncological outcomes, rendering active surveillance a much safer option. In the study by Jain et al. [20], 50% of the low- to intermediate-risk patients had concerning features on their PSMA PET/CT scans. Prostatectomies found that 60% of patients with abnormal PSMA PET/CT results had underlying adverse pathology (upgrading of ISUP GG, EPE, cribriform pattern, or intraductal pattern) which was not detected on mpMRI. The patients who were managed with active surveillance in this study remained on active surveillance. The ability to identify adverse pathology and concerning lesions missed in MRI results appears to demonstrate an additive benefit of PSMA PET/CT compared to the current mpMRI-led active surveillance. However, there are insufficient data to suggest that PSMA PET/CT can replace mpMRI during active surveillance.

PSMA is a transmembrane protein that is upregulated in PCa [25]. Previous studies have demonstrated the correlation of PSMA avidity to the grade of PCa; hence, various PSMA PET SUVmax threshold values have been proposed for the detection of csPCa [26,27,28]. The study by Pepe et al. 2023 [18] and Heetman et al. 2023 [19] utilized a SUVmax cut-off of 4 and 5, respectively, to trigger a PSMA-targeted biopsy. This appears to be congruent with the finding by Xue et al. 2022 [21], where a threshold SUVmax of 5.4 predicted pathological upgrading with 91% specificity and a negative predictive value of 94%. However, there is insufficient evidence at this stage to conclude the optimal SUVmax cut-off that should be used. Based on the experience of the included publications, several possible indications for PSMA-targeted biopsy include investigation of an MRI occult lesion, PSMA avidity discordant with original biopsy results, and the involvement of a MDT whose second opinion may detect PSMA lesions that were missed on the pre-biopsy MRI, as seen in the Heetman et al. study [19]. In addition to SUVmax, the anatomical location of the lesion should be taken into account during assessment as the majority of PCa develops within the peripheral zone. The usage of a scoring system such as the PRIMARY score which incorporates anatomical location and PSMA PET/CT characteristics could improve the assessment of PSMA avid lesions [29].

There is still a lack of consensus on criteria for the selection of intermediate-risk PCa for active surveillance as this cohort consists of a heterogeneous group of patients. Careful selection of intermediate-risk PCa is paramount as it is known to have worse outcomes compared to low-risk PCa on active surveillance [9]. The concern is if the disease has been under-staged during the initial biopsy. The study by Xue et al. [21] demonstrated that SUVmax from PSMA PET/CT can be useful in triaging intermediate-risk PCa patients. PSMA PET/CT was able to differentiate patients with <10% Gleason pattern 4 disease from those with >10% (3.03 versus 4.54, *p* < 0.001). This is particularly useful in patients with ISUP GG 2 prostate cancer. The SUVmax could also differentiate <50% Gleason pattern 4 disease from those with >50% Gleason pattern 4 (3.31 versus 5.51, *p* < 0.001). PSMA PET/CT may have the ability to detect ISUP GG 3 PCa, which was under-staged on biopsy as ISUP GG 2. The ability of median SUVmax to differentiate ISUP GG 2 from ISUP GG 3 was demonstrated in another study evaluating PSMA PET/CT for all patients with suspected PCa [22]. Currently, the widely used D’Amico risk classification of PCa was not made for active surveillance patients as it was originally developed to assess the risk of biochemical recurrence [17]. A new risk stratification specific for PCa patients on active surveillance could be developed to incorporate the SUVmax.

The study by Pepe et al. 2023 [18] demonstrated higher diagnostic accuracy with PSMA PET/CT-targeted biopsies compared to mpMRI-targeted biopsies. These findings are congruent with previous studies that compared PSMA PET/CT-targeted biopsies to transrectal ultrasound-guided biopsies [30] and mpMRI-targeted biopsies [31]. The benefit of improved diagnostic accuracy with PSMA PET/CT is two-fold. Firstly, a PSMA PET/CT-targeted biopsy could detect under-staged prostate cancer in patients who were initially triaged into active surveillance by mpMRI. Secondly, the greater diagnostic accuracy of PSMA PET/CT could potentially translate into a reduction in the number of unnecessary biopsies during active surveillance. However, there is insufficient evidence at this stage to suggest that PSMA-targeted biopsies can replace MRI-targeted biopsies. Interestingly, in the same study by Pepe et al. [18], saturation prostate biopsies detected more csPCa as compared to PSMA- and MRI-targeted prostate biopsies. This finding should be interpreted with caution as it was unclear if these csPCa were mutually exclusive (i.e., if csPCa found on a targeted biopsy was also found or missed on a saturation biopsy). Previously, a meta-analysis demonstrated that combining MRI-targeted biopsies with saturation biopsies resulted in maximized cancer detection during active surveillance [32]. Additionally, the long-term outcomes of active surveillance in men who underwent baseline MRI prior to confirmatory biopsy had half the failure rate of active surveillance and reduced progression to higher-grade cancer when compared to men who only underwent systematic biopsies [33].

The timing of the PSMA PET scans varied between studies and we were unable to reach a conclusion as to the optimal time to perform PSMA PET during active surveillance. In resource-limited centers with minimal and limited access to PSMA PET/CT scanners, PSMA PET/CT could be offered as a once-off scan prior to confirmatory biopsy. This allows for early detection of MRI occult lesions and appropriate staging prior to the continuation of active surveillance. In places where PSMA PET/CT is more widely available, PSMA PET/CT could also be employed selectively during the active surveillance process when there are concerning changes in PSA or mpMRI to assist in deciding whether further investigation with a repeat biopsy is required. This is particularly relevant for patients who wish to continue active surveillance but are averse to the complications associated with a prostate biopsy.

One possible drawback of incorporating PSMA PET scans into active surveillance is the risk of false positive findings outside of the prostate which may result in over-investigation. In the study by Pepe et al. [18], two false positive lesions concerning metastatic disease were seen in the iliac ala and spinal cord on the PSMA PET scans. However, these lesions were not present on further investigation on MRI. The exact percentage and risk for false positive findings outside of the prostate is unknown at this stage.

We appreciate that there are limitations to this systematic review. Firstly, only four studies were identified as suitable for inclusion, and the included study population was heterogeneous, with some studies including only low-risk PCa whilst others included only intermediate-risk PCa. This heterogeneity precluded a meta-analysis. Secondly, two of the studies had small sample sizes and two were retrospective in nature, one of which was a single surgeon experience. Thirdly, the majority of the studies utilized 68Ga-PSMA PET/CT; however, one study included the 18 F DCFPYL PSMA tracer which may have differing properties [34]. Despite these limitations, this is the first systematic review attempting to evaluate the role of PSMA PET in active surveillance. This study highlights a lack of prospective randomized control trial data in this area. Future studies could help elucidate the optimal timing of PSMA PET/CT during active surveillance, comparing PSMA-targeted biopsy to MRI-targeted biopsy, and the influence of PSMA PET/CT on the long-term oncological outcome of PCa patients on active surveillance.

## 5. Conclusions

In conclusion, PSMA PET/CT appears to be a promising tool in the management of low- to intermediate-risk PCa. It can improve our risk stratification and the safety of active surveillance by detecting MRI occult lesions and identifying patients at risk of pathological upstaging. PSMA PET/CT could also play a part in the decision-making of intermediate-risk PCa suitability for active surveillance by identifying patients with high volume Gleason pattern 4 disease and differentiating ISUP GG 3 from ISUP GG 2 disease. However, there is limited evidence to guide the optimal timing of PSMA PET as part of active surveillance and the optimal SUVmax cut-off for identifying csPCa. Further prospective randomized control trials are needed.

## Figures and Tables

**Table 1:** The Quality Assessment of Diagnostic Accuracy Studies (QUADAS-2) assessment of included studies.

	Risk of Bias				Applicability Concerns		
	Patient Selection	Index Test	Reference Standard	Flow and Timing	Patient Selection	Index Test	Reference Standard
Pepe et al., 2023 [18]	Low	Low	Low	Low	Low	Unclear	Low
Heetman et al., 2023 [19]	Low	Low	Unclear	Unclear	Low	Low	Low
Jain et al., 2023 [20]	High	Low	Low	Low	Low	Low	Low
Xue et al., 2022 [21]	High	Unclear	Low	Low	Low	Low	Low

**Table 2:** Study characteristics and outcomes.

Author, Year	Pepe et al., 2023 [18]	Heetman et al., 2023 [19]	Jain et al., 2023 [20]	Xue et al., 2022 [21]
Country	Italy	Netherlands	Australia	Australia
Study period	May 2013 to December 2021	May 2020 to December 2021	January 2019 to October 2022	November 2015 to January 2021
Sample size	40	141	30	220
Inclusion criteria	ISUP 1 + any of the following: ■ life expectancy > 10 years ■ clinical stage T1c ■ PSA < 10 ng/mL, density < 0.20 ■ ≤2 unilateral positive biopsy ■ maximum core % of PCa ≤ 50%	■ Patients with newly diagnosed PCa (<6 months) ■ PSMA PET/CT was performed at a median of 2.0 mo after the start of AS.	■ clinical stage T1-T2 ■ PSA ≤ 15 ■ ISUP GG ≤ 2 (<5% pattern 4) ■ <50% of cores involved. ■ NO cribriform architecture or intraductal carcinoma	All patients with ISUP GG 2 or 3 on biopsy that subsequently underwent PSMA PET
D’Amico classification of included patients	Low-risk PCa only	Low-risk and intermediate-risk PCa	Low-risk and favorable intermediate-risk PCa	All intermediate-risk PCa only
Study design	Prospective Single center, multiple surgeon	Prospective Multiple center	Retrospective Single center, Single surgeon	Retrospective Multiple center
Intervention	PSMA and mpMRI five years from confirmatory biopsy	PSMA PET only after diagnostic biopsy	PSMA PET only after diagnostic biopsy	PSMA PET only after diagnostic biopsy
Pre-planned treatment	AS	AS	AS (37%) or active treatment (63%)	AS (39%) or active treatment (61%)
Tracer	68Ga-PSMA PET/CT	68Ga-PSMA PET/CT	68Ga-HBEDD-11 and 18 F DCFPYL	68Ga-PSMA PET/CT
Median age	63 (range 52–74)	Overall median not available (range 63–73)	64 (IQR 56–68)	Overall median not available (range 62.49–73.84)
Median PSA	4.8 (range 4.5–12.5 ng/mL)	Median not available (range 4.0–7.8)	5.5 (IQR 3.7–6.5)	Median not available (range 4.56–8.60)
Median PSA density	0.15 (range 0.10–0.20)	Median not available (range 0.08–0.17)	Not available.	Median not available (range 0.129–0.260)
Repeat target biopsy	Imaging based cognitive target biopsy ≥ 4 core if PI-RADS score ≥ 3 or SUVmax ≥ 5	Targeted biopsies of ≥3 core if new lesion SUVmax ≥ 4 or MRI lesion showed such high SUVmax	Not specified. But had targeted lesion if concerning lesion on PSMA PET	Target biopsy if MRI has PI-RADS score ≥ 3. No PSMA guided biopsy
Outcome	■ PSMA PET/CT has better diagnostic accuracy in comparison with mpMRI (83.3% vs. 70.2%).	■ PSMA PET picks up MRI occult patient ■ 45 (32%) needed additional PSMA targeted biopsy with 13 (9%) having upgrade in ISUP GG and 1 (0.07%) detected cribiform pattern	■ In patients thought to be suitable for AS, 50% had concerning features on PSMA, and 33.3% had ≥1 adverse pathology on RARP (cribriform pattern, intraductal pattern, extracapsular extension, or upgrade in ISUP GG).	■ SUVmax can predict % of Gleason pattern 4
Limitations	■ Small sample size ■ Scans were done 5 years after confirmatory biopsy, unclear applicability to newly diagnosed patients	■ 7% of patients with intraprostatic lesion with SUVmax ≥ 4 did not undergo repeat prostate biopsy due to various reasons ■ Retrospectively, some PSMA lesion were missed on pre-biopsy MRI	■ Small sample size ■ Single surgeon ■ Retrospective nature	■ Retrospective ■ Non-blinded ■ SUVmax may have varied as scanners across four sites not standardized

Acronyms used: Active surveillance (AS), Multiparametric magnetic resonance imaging (mpMRI), International Society of Urologic Pathology Grade Groups (ISUP GG), Interquartile range (IQR), Prostate Imaging Reporting and Data System (PI-RADS), Maximum Standardized Uptake Value (SUVmax).

## Data Availability

This study only utilizes existing published data.
